# Association of Parental Support with Reduced Stereotypy in Children with Autism Spectrum Disorder: A Cross-Sectional Study

**DOI:** 10.3390/medicina59091667

**Published:** 2023-09-15

**Authors:** Renandro de Carvalho Reis, Isadora Noanda Barbosa Souza, Maria Carolina Rodrigues Dias, Cíntia Maria de Melo Mendes, Kelson James Almeida

**Affiliations:** 1 Department of Medicine, University Center UNINOVAFAPI, Teresina 64073-505, Brazil; 2 Department of Medicine, University Center IDOMED/UniFacid, Teresina 64073-505, Brazil; 3 Pharmaceutical Sciences Graduation Program, Federal University of Piauí, Teresina 64049-550, Brazil; 4 Department of Neurology, Federal University of Piauí, Teresina 64049-550, Brazil

**Keywords:** Autism Spectrum Disorder, parental support, autistic children, social communication, stereotypy

## Abstract

*Background and Objectives*: To analyze the influence of parental presence and use of risperidone on social interaction and apathy among patients with Autism Spectrum Disorder (ASD). *Materials and Methods*: Cross-sectional study in a reference center for patients with ASD in a city in northeastern Brazil. The research was carried out using a sociodemographic questionnaire, the Dimensional Apathy Scale, and the Social Communication Questionnaire (SCQ) with the domains of social interaction, language, stereotypy, and communication. The referred questionnaire was answered by the parents or guardians of the children with ASD according to the DSM V criteria. Data were analyzed via independent *t*-test using the SPSS software version 20. *Results*: Interviews were conducted with 51 parents/guardians of autistic children with a mean age of 8.8 years (±2.95) and a predominance of males, 34 (66.7%). Of this total, 49 (96.1%) of the children attended school; 40 (78.4%) children were on medication, of which 38 (74.5%) were on risperidone. Those children on risperidone had a higher score on the SCQ scale (*p* = 0.049) and on the domain of stereotyped behaviors (*p* = 0.033), which indicated greater impairment. Another statistically relevant variable was the presence of married parents, whereby children who did not have the presence of married parents had a higher average of stereotyped behaviors compared to those who had married parents. *Conclusions*: The results showed differences in the means of social interactions for children on risperidone, especially regarding stereotyped behaviors. However, it is not possible to state whether this difference was due to the use of risperidone or whether they used risperidone precisely because of these behaviors. Also important was that children who had the presence of married parents showed fewer stereotyped behaviors. There was no difference in apathetic behavior between children.

## 1. Introduction

Autism Spectrum Disorder (ASD) is a developmental disorder characterized by difficulties in communication and social interaction and by the presence of repetitive or restricted behaviors and/or interests. Although defined by these main symptoms, the phenotype of patients with ASD can vary greatly, ranging from individuals with severe intellectual disability (ID) and low performance in adaptive behavior skills, to individuals with normal intelligence quotient (IQ). For these patients with ASD, Applied Behavioral Analysis (ABA) therapy becomes a viable and effective indication [1,2,3,4].

In this context, the process of child development and school inclusion must be monitored in its different development indicators, such as aspects of psychomotricity, sensory functions, language, communication, cognition, and socio-adaptive functioning. Aspects to be analyzed concern the development of social interaction and social apathy [5].

Apathy is a clinical syndrome characterized by a reduction in self-initiated, goal-directed activity that is not accounted for by a primary motor impairment or sensory impairment. This is not a unitary syndrome, but it can be manifested in several domains, notably cognition, behavior, emotion, and social interaction [6,7].

Very uninterested and apathetic children, as well as very agitated and unfocused children, may have inadequate social interaction [8]. The pragmatic impairment in children with ASD is believed to be more relevant due to both the inadequacy of social interaction and the impairment of verbal and non-verbal communication (in the absence of recognition of the other and shared attention) in comparison with the performance of children with semantic-pragmatic failures and diagnosed with Specific Language and Speech Disorder [9]. Therefore, social and prosocial behaviors in patients with ASD are often stimulated by using different types of protocols and applied technologies [10,11].

Antipsychotic drugs are commonly used in children and adolescents with ASD. To date, no psychopharmacological medication targeted at the core symptoms of ASD has been approved by the Food and Drug Administration (FDA). However, risperidone as an antipsychotic helps to treat certain common symptoms of the ASD patient, such as aggression towards others, self-injurious behavior, tantrums, and rapid mood swings. It also increases the adaptation of the individual with ASD to the social environment [12].

One of the difficulties when facing the challenges of having a child with ASD is knowledge. As parents are the main caregivers, recognizing the signs and symptoms of ASD is very important to provide the best health care [13]. Taking care of a child with ASD is challenging because it can generate overload and not result in the proper care that the child needs [14], hence the importance of parents being married, as the partner is usually a source of support [15].

Elucidating how different types on the ASD spectrum are associated with personality development may be more helpful than assigning a personality disorder label. On average, ASD patients tend to show less openness, conscientiousness, agreement, and emotional stability than non-autistic people in personality traits. Clarifying the developmental relationships between ASD and personality ensures that subjects are well-understood and supported as early as possible [16]. Therefore, this study aimed to analyze differences in means of social interaction and apathy with medication and social variables among patients with ASD.

## 2. Materials and Methods

### 2.1. Study Design

The cross-sectional study was carried out using the Dimensional Apathy Scale and the Social Communication Questionnaire (SCQ), which serves to investigate types of social interaction, language, and communication. It is answered by parents or guardians of children with ASD diagnosed according to the criteria of DSM V [17].

The sample calculation was performed according to the following formula: *n* = N·Z2·p·(1 − p)/Z2·p·(1 − p) + e2·N − 1 (*n*: calculated sample, N: population, Z: normal variable, p: real probability of the event, e: sampling error); this resulted in 51 patients. The research was carried out in a reference center for the treatment of special children in a city in northeastern Brazil.

### 2.2. Study Variables

Sociodemographic variables involved age, gender, education, age at diagnosis of symptoms, whether the patient attended school regularly, use of medication, treatment with multi-professional therapy (at least twice a week, at least with Speech Therapy or Psychology professionals), diagnosis of ASD (level 1, or 2 or 3—according to the levels defined by the DSM V [18]), parents’ schooling, and whether the parents were married or not (stable union without formal marriage was considered as marriage).

Apathy was assessed using an appropriate scale developed by Starkstein et al. [18] and translated into Brazilian Portuguese [19]. Scores range from 0 to 42 points, and higher values suggest more severe symptoms. This scale has 14 questions, each with a score from 0 to 3, which proposes the investigation of different aspects of apathetic symptoms. The scale produces a dimensional assessment of apathetic symptoms, without accurately allowing a categorical diagnosis of apathy.

The Social Communication Questionnaire (SQC) was validated for the Brazilian Portuguese language by Sato et al. [20]. The instrument consists of 40 questions covering four major domains, namely, social interaction (20 items: 17, 19, 21–23, 26–40), language (6 items: 3–7), stereotypies (7 items: 8, 10–14, 16, 18), and behaviors (6 items: 2, 9, 15, 20, 24–25). The first SCQ question is not scored. The remaining 39 items are scored either 0 or 1, resulting in a total SCQ score between 0 and 39 [21].

### 2.3. Recruitment Procedures

Guardians of child patients with a clinical diagnosis of ASD were included; the age of patients was between 5 and 11 years old, and patients were registered and attended a reference center for special children in the city of Timon, state of Maranhão, northeastern Brazil. Exclusion criteria were the absence of a person responsible for authorizing and answering the research questionnaire, those who chose not to participate, and one of the parents with some cognitive impairment or intellectual disability that prevented him/her from answering the questionnaires.

### 2.4. Assessment Details

The research began after applying the exclusion and inclusion criteria and the signing of the Informed Consent (IC) by the parents or guardians of the participants. Then, those responsible answered the questionnaires about apathy and Social Interaction. In the end, the results obtained were evaluated.

### 2.5. Ethical Procedures

The research project was developed in accordance with Resolution 466/2012 of the National Health Council, which defines the guidelines and regulatory standards for research, mainly involving human beings. The project was approved by a local research local ethics committee according to CAAE number 59992922.7.00005210. The research was only carried out under the signature of the IC.

### 2.6. Statistical Analysis

Data were organized in Excel spreadsheets and later analyzed using the Statistical Package for the IBM Social Sciences (SPSS) software version 20 (IBM Inc., Chicago, IL, USA). Data were organized into absolute and relative frequencies with the result of the sociodemographic questionnaire, perception of apathy, and social interaction. An independent *t*-test was applied, and for nonparametric data, the Mann–Whitney U test was used, to compare mean apathy scores and social interactions that increased with the presence of married parents and risperidone use. Differences were considered significant for *p* < 0.05 and 95% confidence interval.

## 3. Results

This study analyzed only the child population, which resulted in a total sample of 51 children among the 124 registered at the reference center. Table 1 details the sociodemographic variables, medication use, and clinical staging of patients with ASD. An independent *t*-test was applied when the variables were continuous. The mean age was 8.1 years (±2.95) and the initial symptoms started at 2.47 years (±1.59). The male gender was the most frequent, and 66.7% children had married parents. Only two children had severe ASD and did not attend school.

Table 2 lists the results of the comparison between children taking risperidone and not taking it. Children on risperidone had a higher overall SCQ score than those not taking it (*p* = 0.049). Within the SCQ domains, children on risperidone had a higher score on stereotyped behaviors (*p* = 0.033).

Table 3 presents the results of the comparison between children who had married parents and those who did not. Children who did not grow up without the presence of married parents had statistically higher scores of stereotyped behaviors (*p* = 0.019), one of the domains of the SCQ questionnaire.

## 4. Discussion

Our findings show that the group on risperidone had higher social communication scores. Hartmann et al. [22] demonstrated that at lower levels of social skills, in which alternative communication strategies are limited, there are indications that self-injurious behavior (SIB) is more likely to become socially accentuated. Therefore, such medication is effective and well tolerated for this self-injurious behavior [23].

Of the 40 children on medication, 38 were taking risperidone. Risperidone is an approved and safe medication for use in children with severe ASD symptoms, especially irritability and behavioral changes [24]. Stereotyped behavior is one of the symptoms that patients with ASD who use antipsychotics, especially risperidone, in addition to other behaviors such as hyperactivity and irritability [25].

In social communication, stereotyped behavior was the significantly relevant variable. Komatsu et al. [26] found that impaired attention switching, a symptom of ASD, positively affects stereotype endorsement, which impairs social communication. When due attention/psychological treatment is not given, cases of stereotypy can be aggravated.

As observed in Table 2, social communication as a form of social behavior was a statistically significant variable. Behavioral aspects can influence the development of children with ASD. For that, the biopsychosocial aspects of development are considered. Inclusive behavior teaching programs that involve different areas of development are also socially relevant: language, socialization, self-care (eating, drinking, dressing, bathing independently), and academic skills [27].

Table 3 shows the importance of the presence of the parents for the social development of the autistic child because children without the presence of both parents had a greater commitment in the stereotyped behavior. The presence of both parents in a stable relationship or married is necessary for the better development of the children. The presence of parents can assist in the development of appropriate interventions to further support the children [28]. In several aspects, the presence of parents is necessary, as demonstrated by Crane et al. [29] with autistic parents who found it easier to talk to their children about of patient with ASD and how to face life.

Parents and family have a significant influence on children’s developmental conditions and dysfunction is strongly associated with psychopathy in children [30]. Lung [31] categorized “single parents” as a high-risk family trait for patient with ASD, with a 1.05 and 1.21 chance of being diagnosed with ASD and ADHD, respectively. Likewise, a study has shown that better family cohesion and adaptability improve the influence of the family environment on children’s ADHD symptoms [32].

Parental behavior can also enhance the cognitive and social development of ASD in addition to playing an important role in many interventions [33]. Several interventional studies with the active participation of parents [34,35,36,37,38] have already demonstrated the importance for their children to have good development in communication and social interaction. One of these interventions that demonstrates the importance of parents for autistic children is the study by Nowell et al. [39], who also applied the SCQ; an intervention assisted by both parents and children was performed, which demonstrated an increase in knowledge of the concept of social communication, which helps parents to better deal with their children’s social interactions.

One of the limitations of the present study is that it presents information obtained at a given time, for these reasons, it may be subject to temporal and sociocultural influences. Another limitation was the fact that apathy is a symptom that is difficult to diagnose and that it can be even more difficult to diagnose when there is severe cognitive impairment, but in this study, there were only two patients with ASD level 3. Symptoms such as difficulty in maintaining a fixed gaze, which is very common in ASD, may contribute to the diagnosis of apathy. Confounding biases regarding disease severity and indication for Risperidone may explain some of the associations when evaluating this dependent variable.

## 5. Conclusions

The results showed differences in means for children on risperidone in social interactions, especially with regard to stereotyped behaviors. However, it is not possible to state whether these behaviors persisted due to the use of risperidone or whether they used risperidone precisely because of these behaviors. As this was a cross-sectional study, risperidone was more likely applied to the most severe patients, who had worse scores on the SCQ scale. However, the finding is just an association whose probable severity bias was confounding, as previously mentioned.

Another relevant factor was that children who had the presence of married parents showed fewer stereotyped behaviors. This point is associated with the diagnosis of children having the presence of parents in the same house can help in the development of social behaviors. However, this study does not suggest that single parents are unable to care for their children.

## Figures and Tables

**Table 1:** Demographic distribution of children and parents.

Variables	*n* or Mean	Percentage	SD
Idade	8.1	-	2.95
Start of symptoms	2.47	-	1.59
Gender			-
Male	34	66.7	
Female	17	33.3	
Attends school			-
Yes	49	96.1	
No	2	3.9	
Use of medication			-
Yes	40	78.4	
No	11	21.6	
Psychopedagogue			-
Yes	33	64.7	
No	18	35.3	
Speech therapist			-
Yes	39	76.5	
No	12	23.5	
Psychologist			-
Yes	42	82.4	
No	9	17.6	
ASD diagnosis			-
Mild—level 1	22	43.2	
Moderate—level 2	27	52.9	
Severe—level 3	2	3.9	
Married parents			-
Yes	34	66.7	
No	17	33.3	

SD = standard deviation; *n* = total.

**Table 2:** Comparison of children on the use of risperidone and apathy and social communication scales.

Variables	Risperidone Use				*p* *
	Yes		No		
	m	SD	m	SD	
Apathy	19.13	3.5	19	3.96	0.91
Social communication	18.92	4.56	16.08	3.77	0.049
Abnormal language	1.55	0.86	1	1	0.061
Stereotypies	4.08	1.51	3	1.58	0.033
Communication	3.32	1.56	3	1.41	0.523
Social interaction	9.97	3.28	9.08	3.35	0.401

m = mean; SD = standard deviation. * independent *t*-test or Mann–Whitney U test.

**Table 3:** Marital status of the parents.

Variables	Married Parents				*p* *
	Yes		No		
	m	SD	m	S	
Apathy	18.65	3.53	20	3.62	0.207
Social communication	17.85	4.43	18.88	4.74	0.448
Abnormal language	1.29	0.8	1.65	1.12	0.2
Stereotypies	3.44	1.44	4.53	1.66	0.019
Communication	3.21	1.49	3.29	1.61	0.847
Social interaction	9.91	3.31	9.41	3.3	0.613

m = mean; SD = standard deviation. * Independent *t*-test or Mann–Whitney U test.

## Data Availability

The data presented in this study are available within the article.
